# Semi-Mechanistic Modeling of HY-021068 Based on Irreversible Inhibition of Thromboxane Synthetase

**DOI:** 10.3389/fphar.2020.588286

**Published:** 2020-11-30

**Authors:** Ping Li, Jie Huang, Donghao Geng, Peihua Liu, Zhaoxing Chu, Jianjun Zou, Guoping Yang, Li Liu

**Affiliations:** ^1^ Center of Pharmacokinetics and Metabolism, School of Pharmacy, China Pharmaceutical University, Nanjing, China; ^2^ Center of Clinical Pharmacology, The Third Xiangya Hospital of Central South University, Changsha, China; ^3^ Department of Clinical Pharmacology, Nanjing First Hospital, Nanjing Medical University, Nanjing, China

**Keywords:** pharmacokinetic/pharmacodynamic model, platelet aggregation rate, HY-021068, pharmacokinetic, thromboxane A_2_

## Abstract

**Background:** HY-021068 [4-(2-(1H-imidazol-1-yl) ethoxy)-3-methoxybenzoate], developed by Hefei Industrial Pharmaceutical Institute Co., Ltd. (Anhui, China), is a potential thromboxane synthetase inhibitor under development as an anti-platelet agent for the treatment of stroke. A semi-mechanistic pharmacokinetic/pharmacodynamic (PK/PD) model was developed to characterize the PK of HY-021068 and its platelet aggregation inhibitory effect in beagle dogs.

**Method:** Beagle dogs received single oral administration of 2.5 mg/kg HY-021068 or consecutively oral administration of 5 mg/kg HY-021068 once daily for 7 days. The plasma concentration of HY-021068 and the platelet aggregation rate (PAR) were determined by liquid chromatography tandem-mass spectrometry (LC‐MS/MS) assay and a photometric method, respectively. The PK/PD data was sequentially fitted by Phoenix NLME. The PK/PD parameters of HY-021068 in beagle dogs were estimated by 2.5 and 5 mg/kg dosing on the 1st day, and then used to simulate the PAR of HY-021068 on the 7th day after 5 mg/kg dosing daily.

**Result:** A one-compartment model with saturable Michaelis-Menten elimination was best fitted to the PK of HY-021068. A mechanistic PD model based on irreversible inhibition of thromboxane synthetase was constructed to describe the relationship between plasma concentration of HY-021068 and PAR. Diagnostic plots showed no obvious bias. Visual predictive check confirmed the stability and reliability of the model. Most of PK/PD observed data on the 7th day after 5 mg/kg dosing fell in the 90% prediction interval.

**Conclusion:** We established a semi-mechanistic PK/PD model for characterizing the PK of HY-021068 and its anti-platelet effect in beagle dogs. The model can be used to predict the concentration and PAR under different dosage regimen of HY-021068, and might be served as a reference for dose design in the future clinical studies.

## Introduction

Stroke is the most common cause of mortality worldwide and is the second leading cause of long-term disability (Sila and Schoenberg, 2011; Alkagiet et al., 2018). Arachidonic acid (AA) is metabolized to TXA_2_ by cyclooxygenase (Cox) and thromboxane synthase (TXS). TXA_2_ is a potent platelet aggregation activator, which contributes to an increase in cytosolic Ca^++^ concentration, further activating calmodulin-dependent myosin light chain phosphorylation and diacylglycerol-dependent cytosolic pleckstrin phosphorylation to induce platelet aggregation (Cho et al., 2011). Anti-platelet drug, such as aspirin, indomethacin, and triflusal, can reduce TXA_2_ formation by inhibiting the activity of COX or TXS, which have a protective effect against cerebral infarction (Mayr and Jilma, 2006; Murdoch and Plosker., 2006; Shin et al., 2018).

**FIGURE 1:**
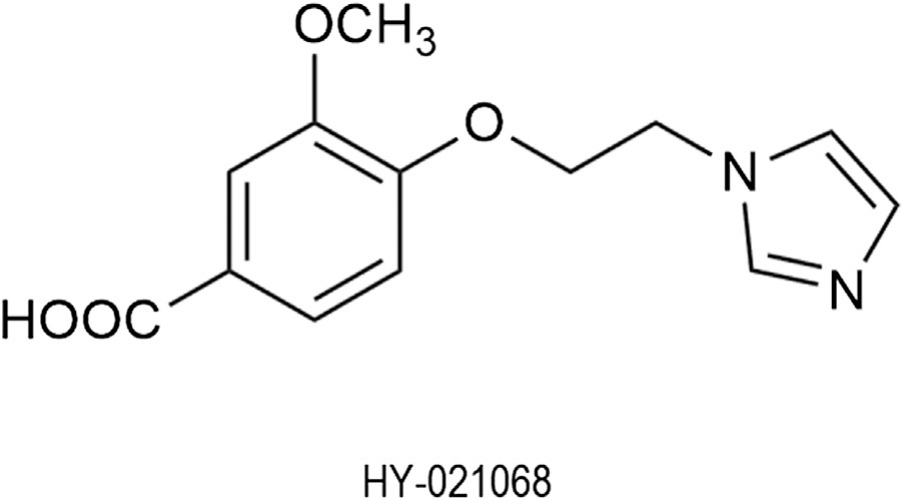
Chemical structure of HY-021068.

HY-021068 (4-(2-(1H-imidazol-1-yl) ethoxy)-3-methoxybenzic acid) (Figure 1) is a promising Class I new drug as an anti-platelet agent under phase I clinical trial developed by Hefei Industrial Pharmaceutical Institute Co., Ltd. (Anhui, China), which is an analog of Dazoxiben (a TXS inhibitor) (Vittorio et al., 1984). It showed protective effect on focal cerebral ischemia and reperfusion injury through inhibiting platelet aggregation. Previous study (Zhou et al., 2014; Yang et al., 2015) indicated that HY-021068 treatment could improve performance in neurological deficit and the Y-maze test as well as reduce the infarct volume in ischemia-reperfusion rats induced by middle cerebral artery occlusion. Incubation of HY-021068 (3 × 10^−5^ and 10^−5^ M) significantly reduced TXB_2_ (stable metabolite of TXA_2_) formation in rat brain microvascular endothelial cells after hypoxia/reoxygenation treatment (Zhou et al., 2014). HY-021068 can inhibit platelet aggregation induced by adenosine 5′-diphosphate or U46619 in a dose-dependent manner *in vivo* and *in vitro* (Yang et al., 2015). The results indicated that the anti-platelet aggregation effect of HY-021068 was strongly correlated with the inhibition of TXA_2_ formation. AA was widely used as platelet agonists (Pyo et al., 2007; Andrioli et al., 2015; Olivier et al., 2016) in the platelet aggregation test. For example, AA-stimulated platelet aggregation was used to evaluate the anti-platelet activity of aspirin (Temperilli et al., 2015) and YS-49, YS-51 (Pyo et al., 2007). Therefore, AA was selected as a platelet aggregation stimulator to evaluate the anti-platelet aggregation activity of HY-021068 in our study.

Pharmacokinetic/pharmacodynamic (PK/PD) model has been widely applied in preclinical and clinical drug research (Liu et al., 2011; Yun et al., 2014; Li et al., 2016). PK/PD model is established by a series of equations to quantify the behavior and action of the drug *in vivo*. A well-constructed PK/PD model can help people better understand the time-course of drug efficacy and provide reference for future experiments and trials.

Platelet aggregation rate (PAR) is widely used for assaying platelet function (Mckenzie et al., 1999) and has been the pharmacodynamic indicator of many PK/PD models for anticoagulants (Yun et al., 2014; Jiang et al., 2016). The PK/PD studies of antiplatelet drugs via modeling have been reported previously (Woo et al., 2002; Hochlolzer et al., 2005; Lee et al., 2012; Ruzov et al., 2015). However, none of them share the same mechanism with HY-021068. To quantitatively assess the PK/PD properties of HY-021068 based on its inhibitory effect of TXS, we established the semi-mechanistic PK/PD model to mathematically describe the relationship between the PK of HY-021068 and the PAR.

## Method

### Reagents and Materials

HY-021068 (purity 99.68%) was provided by Hefei Industrial Pharmaceutical Institute Co., Ltd. (Anhui, China). Terazosin internal standard (IS) was purchased from National Institutes for Food and Drug Control (Beijing, China). Aspirin enteric-coated tablets were purchased from CSPC Pharmaceutical Co., Ltd. Methanol and acetonitrile of high-performance liquid chromatography (HPLC) grade were obtained from Merck (Darmstadt, Germany). All the other reagents were analytical grade. Water was purified using a Milli-Q Ultrapure Water System (Millipore, Bedford, MA, United States).

### Animals

Twenty-four adult beagle dogs (12 male and 12 female, weighing 8–10 kg) were purchased from Yizheng an Li Mao Biotechnology Co., Ltd. They were housed in an environmentally controlled room temperature and humidity on a 12 h light/dark cycle. Water and food were provided ad libitum. All animal experiments were approved by the Animal Ethics Committee of China Pharmaceutical University (No. CPU-PCPK-1622010751).

### Experimental Design

Twenty-four beagle dogs were randomly divided into four groups, with six dogs in each group (half male and half female). I group, which received a single oral dose of 2.5 mg/kg HY-021068. About 1 ml blood samples were collected from the cephalic vein of the forelimb at pre-dose and at 0.0833, 0.25, 0.5, 0.75, 1, 1.5, 2, 3, 4, 6, 8, 10, 12, 14 h after the drug administration. The collected blood samples were divided in two aliquots. One aliquot was used to determine the concentration of HY-021068. The other was used for the platelet aggregation test. II group, which received 5 mg/kg HY-021068 once daily for consecutive 7 days. After the first and last drug administration, blood samples were collected as the same time points as before. III group (blank control), which received the same volume of vehicle (0.5% sodium carboxymethycellulose). Blood samples were collected as the same time points as before. Shuldiner Blood samples were collected at pre-dose and at 0.25, 0.5, 0.75, 1, 1.5, 2, 3, 4, 6, 8, 10, 12 h after aspirin administration for the platelet aggregation test.

### Platelet Aggregation Test

One aliquot of the blood sample collected from six beagle dogs in each group at each timepoint was used for platelet aggregation test. The anti-platelet activity of HY-021068 was evaluated by platelet aggregation test according to previous studies (Shuldiner et al., 2009; Andrioli et al., 2015; Olivier et al., 2016). Platelet-rich plasma (PRP) was isolated by centrifugation of blood samples at 160 g for 10 min. The supernatant was PRP and the remaining specimen was further centrifuged at 2000 g for 10 min to obtain platelet-poor plasma (PPP). The platelet counting and platelet aggregation were both measured by LBY-NJ4 platelet aggregation analyzer (Precil, Beijing, China). The platelet concentration was adjusted to 20 × 10^4^/µl for PRP before aggregation analysis. 100% transmission values were calibrated using PPP samples. The measurements were performed by adding 10 µl AA (1 mM) to 200 µl PRP in a transparent silica tank maintained at 37°C with agitation at 1,000 rpm. Platelet aggregation was monitored for 5 min. The maximum PAR was the maximum percent change in optical density with PRP after adding aggregation agonist. The results of platelet aggregation were expressed as the maximum PAR.

### Model Development

**FIGURE 2:**
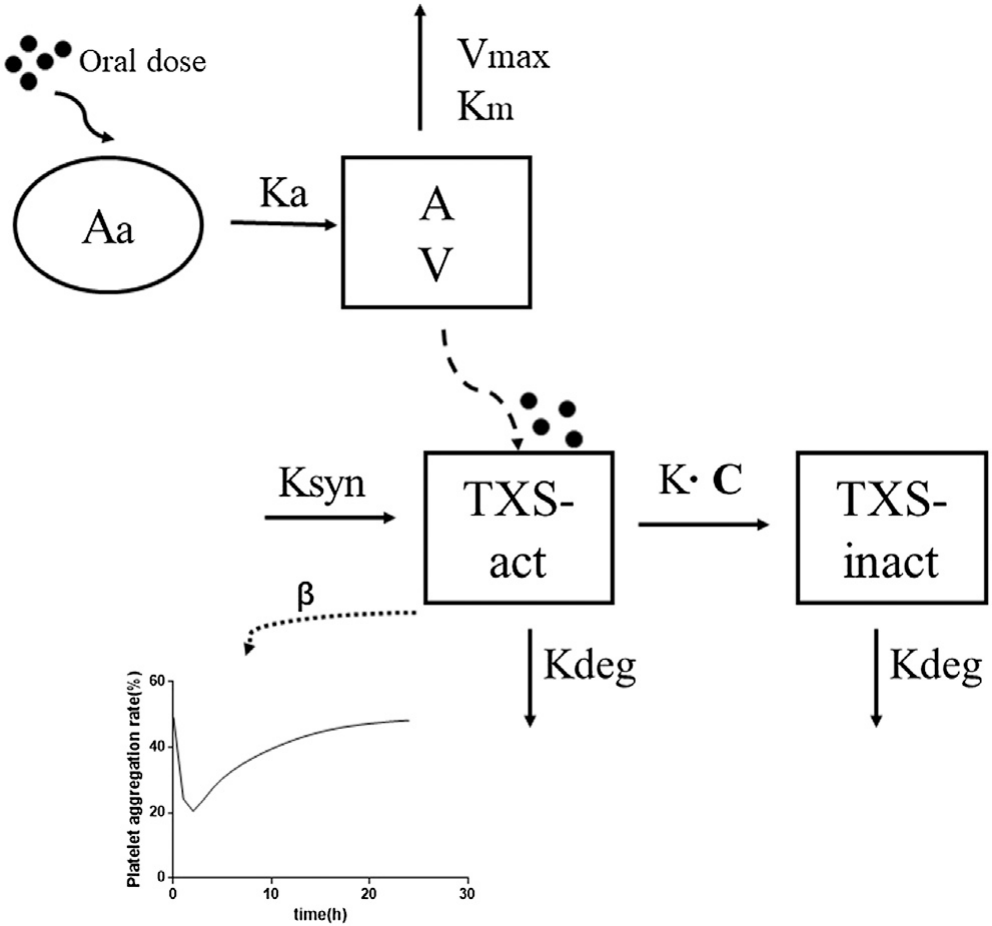
The schematic diagram of pharmacokinetic/pharmacodynamic model of HY-021068.

The PK profiles of HY-021068 was first analyzed by non-compartmental analysis using Phoenix WinNonlin software. The dose-normalized area under the curve (AUC) values were compared by Student’s t test. The PK/PD analysis was performed using Phoenix NLME (version 7.0, Certara, Co., Princeton, NJ, United States). All parameters were estimated using NLME’s first-order conditional estimated extended least squares (FOCE-ELS) method. The PK/PD parameters of HY-021068 in beagle dogs were estimated by 2.5 and 5 mg/kg dosing on the 1st day, and then used to simulate the PAR of HY-021068 on the 7th day after 5 mg/kg dosing daily. The schematic diagram of PK/PD model was shown in Figure 2. The details of PK/PD model were as follows.

### Pharmacokinetic Model

HY-021068 concentration-time profiles were described by compartmental models in beagle dogs. One-, two-, three-compartment model with first-order absorption and saturable Michaelis-Menten elimination were tested to fit the pharmacokinetic data. The equations for final selected model are shown below:

$$\frac{dA_{a}}{\mathrm{dt}}=-K_{a}\times \;A_{a}$$

$$\frac{dA_{1}}{\mathrm{dt}}=-K_{a}\times A_{a}-\frac{V_{\max}\times C}{K_{m}+C}$$

$$C=A_{1}/V$$

Where A_a_ and A_1_ are amounts of HY-021068 in the absorption and central compartments, respectively. V_max_ and K_m_ are the Michaelis-Menten constants for elimination process.

### Pharmacodynamic Model

A sequential PK-PD modeling approach (Zhang et al., 2003) was applied to describe the time course of concentration of HY-021068 and PAR. Irreversible inhibitory effect model was used based on its pharmacology mechanism. We assume 1) HY-021068 reduced TXA_2_ entirely by inactivating TXS. 2) PAR was directly proportional to the amount of active TXS.

$$\frac{{\text{dE}}_{\text{act}}}{\text{dt}}={\text{K}}_{\text{syn}}-{\text{K}}_{\text{deg}}\times {\text{E }}_{\text{act}}-K\times \text{C}\times {\text{E}}_{\text{act}}$$

$$\mathrm{PAR}=\beta \times \;\frac{{\text{E}}_{\text{act}}}{{\text{E}}_{0}}\times 100\text{\%}$$

$$E_{0}=\frac{{\text{K}}_{\text{syn}}}{{\text{K}}_{\text{deg}}}$$

Where *β* is a coefficient corresponding to HY-021068 efficacy, E_act_ is the amount of active TXS *in vivo*, K_syn_ is the zero-order synthesis rate and K_deg_ is a first-order elimination rate constant of TXS. HY-021068 inactivates platelet TXS irreversibly with the second-order rate constant, K. E_0_ is the amount of active TXS in blood before HY-021068 administration.

### Random Effect Model

The intra-individual variability for PK data between observed and predicted values was best described by proportional model.

CObs=C×(1+Ceps)

The intra-individual variability for PD data between observed and predicted values was best described by a mixed error model as follows.

$$\mathrm{PARObs}=\mathrm{PAR}+\mathrm{PAReps}\times \sqrt{1+{(\mathrm{multStdev}/\mathrm{sigma}\left(\right))}^{2}}$$

The inter-individual variablity model used log-additive model.

$$\;P_{i}\times \mathrm{tvP}\times \exp\left(\eta_{i}\right)$$

Where CObs and PARObs are the observed values for PK and PD data, and Ceps and PAReps are the residual error assumed to be normally distributed with mean 0 and variance of $\sigma^{2}$. Multiplicative sigma is a fixed effect called multStdev. “sigma()” is a built-in function which means the estimate of SD of PAReps. P_i_ is the parameter for the *i*th individual. TvP is the typical value of the parameter in the population, and ηi is the interindividual variability of the parameter with mean 0 and variance of $\omega^{2}$.

### Model Evaluation and Validation

General goodness-of-fit was evaluated by diagnostic plots, such as observed HY-021068 plasma concentration or PAR vs. predicted concentration or PAR, conditional weighted residuals (CWRES) vs. predicted values or time.

Final population PK/PD models were evaluated using a simulation that involves 1,000 data sets from the final model parameters, 90% confidence intervals were computed and presented for visual inspection along with the original dataset. The predictive ability of our model was further assessed in the remaining dataset gained from 5 mg/kg oral dosing for consecutive 7 days.

### LC-MS/MS Analysis of HY-021068

The LC–MS/MS system consisted of a Shimadzu Nexera HPLC system and a triple-quadrupole tandem mass spectrometer of Shimadzu MS-8030 system (Kyoto, Japan). An aliquot of 5 μL extracted sample was performed at 40°C using a Shim-pack VP-ODS analytical column (2.0 × 150 mm, 5 μm, Shimadzu, Japan) for separation. The mobile phase consisted of Milli-Q water containing 0.1% formic acid (mobile phase A) and methanol (mobile phase B) at a flow rate of 0.2 ml/min. The gradient elution was: 0–0.2 min, 12% B; 0.2–0.4 min, 12–60% B; 0.4–4.5 min, 60% B; 4.5–4.7 min, 60–12% B; 4.7–8.5 min,12% B (v/v). The last 3.8 min was held to re-equilibrate. The overall run time to determine the concentration of HY-021068 was 8.5 min.

The mass spectrometer used electrospray ionization in the positive ion mode, and the detection of the ions was obtained in the multiple reaction monitoring (MRM) mode. It monitored the transitions of protonated precursor ion at m/z 263.10→68.10 for HY-021068 and m/z 388.15→290.20 for internal standard (IS, terazosin). Mass spectrometric conditions for analysis were optimized as follows: 4.5 kV interface voltage, 1.72 kV detector voltage, 300°C desolation temperature, 400°C heat block temperature, 3 L/min nebulizing gas flow (nitrogen), 15 L/min drying gas flow (nitrogen), and 230 kPa collision induced dissociation (CID) gas pressure (argon). Compounds parameters, Q1 Pre Bias, collision energy (CE) and Q3 Pre Bias, were −23, −38 and −14 V for HY-021068 and −13, −30 and −18 V for IS, respectively.

Stock solutions of both HY-021068 and IS were prepared by dissolving required amounts of reference standard in methanol at a 1.0 mg/ml concentration. The stock solution was serially diluted to working solution of desired concentrations with methanol. The IS working solution was performed by diluting the IS stock solution (1 mg/ml) to final concentration of 1,000 ng/ml with methanol. Calibration standards were prepared by adding the appropriate amounts of the standard solutions into blank plasma giving concentrations of 7.8, 15.6, 31.25, 62.5, 125, 250, 500 and 1,000 ng/ml for HY-021068. For method validation, three concentration levels (15.6, 125 and 800 ng/ml) were prepared for the quality control (QC) samples as the low, medium and high concentration.

An aliquot of 50 μl plasma and 10 μl of IS (1,000 ng/ml) were added into a 1.5 ml centrifuge tube. The mixture was extracted by 1 ml of ethyl acetate, with vertexing for 10 min. After centrifugation at 11,350 g for 10 min, the supernatant (800 μl) was quantitatively transferred into a clean 1.5 ml centrifuge tube and then evaporated to dryness in a vacuum evaporator (Thermo, Waltham, Massachusetts, United States). The residue was reconstituted in 500 µL mobile phase followed by ultracentrifugation at 26000 g for 10 min. An aliquot of 5 µL supernatant was injected into the LC-MS/MS system for analysis.

### Bioanalytical Method Validation

According to FDA guidance (FDA, 2018)for bioanalytical method validation, a full validation was performed for the concentration measurement of HY-021068 in beagle dogs.

#### Selectivity

Selectivity was investigated by the comparison of blank plasma from six individual beagle dogs to the corresponding spiked plasma samples to exclude interference of endogenous substances and metabolites. No obvious interferences from endogenous plasma substances were observed at the retention time of the analytes or IS.

#### Linearity and Lower Limits of Quantification

Three calibration curves of HY-021068 were performed with eight concentrations (7.8, 15.6, 31.25, 62.5, 125, 250, 500 and 1,000 ng/ml). The linearity of each calibration curve was determined by plotting the peak area ratio (*y*) of analytes to IS vs. the corresponding concentration (*x*) of analytes with weighted (1/*x* ^2^) least square linear regression. The LLOQ of HY-021068 was defined as the lowest concentration plasma level on the calibration curves (7.8 ng/ml), at which the analyte should be quantified with acceptable accuracy (80–120%) and precision (≤20%).

Calibration curve showed good linearity at 7.8–1,000 ng/ml for HY-021068. The mean (±SD) regression equation from three replicate calibration curves was:

$$Y=\left(0.0094\pm 0.0003\right)\times X+\left(0.027\pm 0.0047\right)\left(R^{2}\geq 09969\right)$$

where Y was the ratio of peak area of HY-021068 to IS, and X was the HY-021068 concentration. The LLOQ of HY-021068 was 7.8 ng/ml. Data for lower limits of quantifications (LLOQ) are shown in Table 1.

**TABLE 1:** The intra- and inter-day precision and accuracy of analytes (3 days, five replicates per-day).

Spiked conc. (ng/ml)	Inter-day (n = 15)			Intra-day (n = 5)			
	Measured conc. (ng/ml)	RSD (%)	Accuracy (%)	Measured conc. (ng/ml)	RSD (%)	Accuracy (%)	
7.8	7.7 ± 0.5	6.52	98.68	7.54 ± 0.51	6.79	96.68	
15.6	15.94 ± 0.89	5.56	102.04	16.33 ± 0.84	5.17	104.48	
125	127.28 ± 5.86	4.6	101.82	132.85 ± 5.17	3.89	106.28	
800	759.81 ± 33.91	4.46	94.98	787.41 ± 18.98	2.41	98.43	

#### Precision and Accuracy

The intra-day precision and accuracy of the method were assessed by analyzing the QC samples ﬁve times on a single day, and the inter-day precision and accuracy were estimated by determining the QC samples over three consecutive days. Relative standard deviation (RSD) and relative error (RE) were used to express the precision and accuracy, respectively. The intra- and inter-day precision should not exceed 15% for the QC samples and accuracy should be within ±15% for the QC samples. The data for accuracy and precision are shown in Table 1. These results demonstrated that the method was reliable and reproducible.

#### Matrix Effect

The matrix effect was evaluated by comparing the analyte peak areas resolved in the blank extracted plasma samples with the same pure reference standard solutions prepared in the reconstitution solution.

The matrix effects of the analytes derived from QC samples were 98.44 and 107.46% for low and high concentration. The results indicated that no apparent matrix effect was observed.

#### Stability

The long-term stability test of analytes in beagle dog plasma was assessed by analyzing the QC samples at low and high concentration levels (n = 5) stored at −80°C for 15 days.

Results of long-term stability are shown in Table 2. The results showed good stability under the storage condition.

**TABLE 2:** The long-term (15 days) stability of analytes.

Spiked conc. (ng/ml)	Stability (n = 5)		
	Measured conc. (ng/ml)	RSD (%)	Accuracy (%)
15.6	14.31 ± 1.85	12.9	91.73
800	837.59 ± 109.99	13.13	104.7

#### Dilution Integrity Test

Concentrations of the HY-021068 obtained from beagle dog plasma may be higher than the calibration range used for the validation analysis. In such cases, dilution integrity experiment should be investigated to analyze higher analyte concentrations above upper limit of quantification (ULOQ). Dilution of samples should not interfere with the accuracy and precision. The test was carried out at eight times the ULOQ concentration. Five replicates each of 1/10 concentration were prepared and their concentrations were calculated by applying the dilution factor 10 against the freshly prepared calibration curve.

The dilution integrity test was determined by measuring the accuracy and precision for samples which underwent ten times dilution with blank beagle dog plasma. The accuracy was 100.36%, representing that a ten times dilution with blank beagle dog plasma was stable.

## Result

### Pharmacokinetics

**FIGURE 3:**
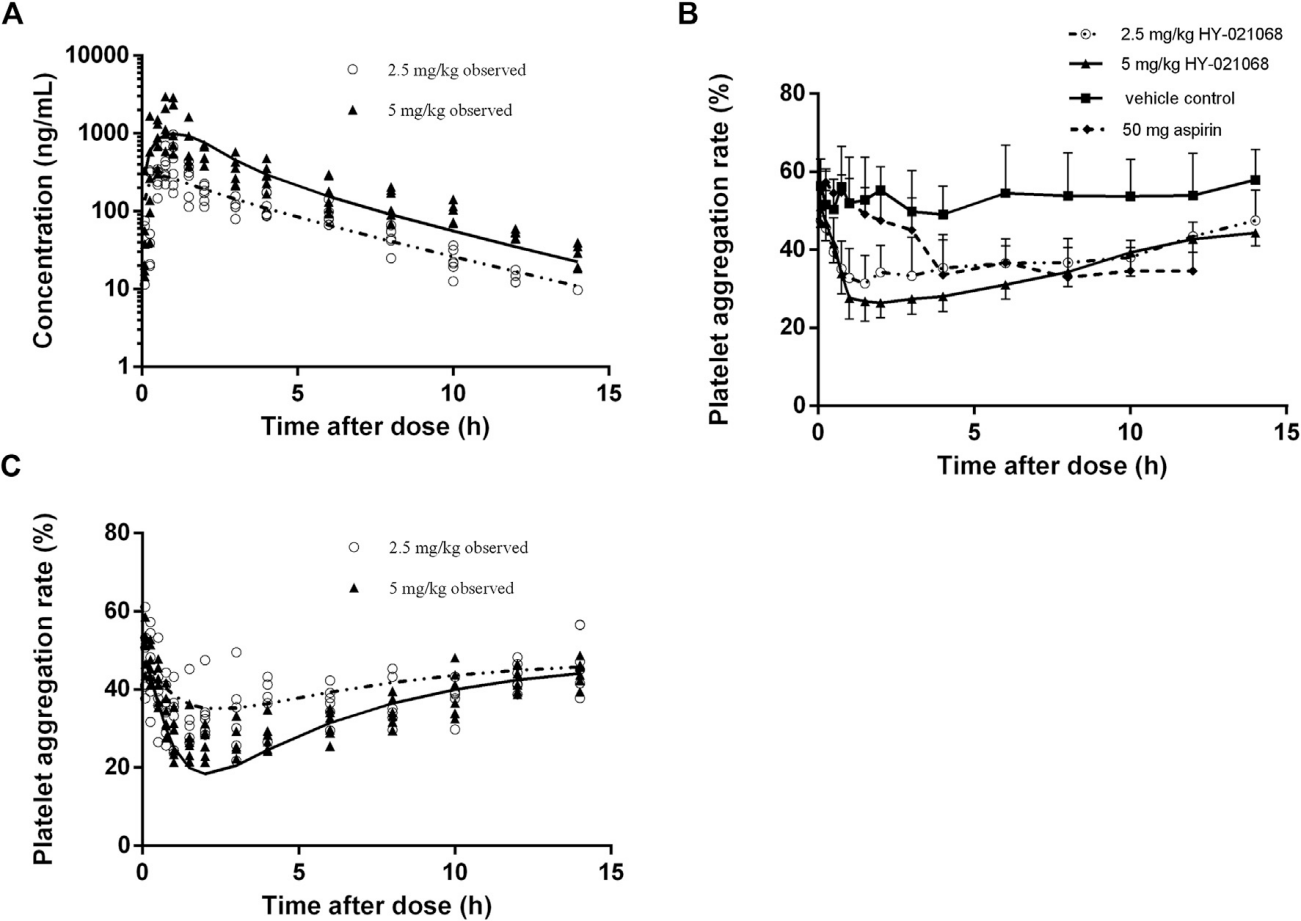
Observed plasma HY-021068 concentration **(A)** and platelet aggregation rate (PAR) **(C)** after a single oral dose of 2.5 mg/kg (open circles, n = 6) and the first administration of multiple doses of 5 mg/kg HY-021068 (triangles, n = 6) to beagle dogs. Curves depict population fittings. (Solid line: 2.5 mg/kg; dashed line: 5 mg/kg). **(B)** Time course of PAR in beagle dogs after oral administration of 2.5 mg/kg HY-021068 (open circles, mean ± SD, n = 6), 5 mg/kg HY-021068 (triangles, mean ± SD, n = 6), 50 mg aspirin (diamond, mean ± SD, n = 6) or vehicle (square, mean ± SD, n = 6).

Noncompartmental analysis of the PK profiles indicated nonlinear PK in the beagle dogs. Dose normalized AUC values after a single oral dose of 2.5 and 5 mg/kg HY-021068 were 0.030 ± 0.008 min × kg/ml and 0.046 ± 0.011 min × kg/ml respectively (*p* = 0.017), so saturable elimination was assumed. Inclusion of more compartment did not improve the model fit. The pharmacokinetic of HY-021068 was well described using a one-compartment model with saturate elimination. Model fitting and observed plasma concentration following oral dosing was shown in Figure 3A and the parameters estimates were presented in Table 3. The inter-individual variability (*ω*) with high shrinkage value (>0.4) was excluded from the PK model to avoid over-parameterization.

**TABLE 3:** Population PK results of oral administration of HY-021068 to beagle dogs.

Parameter	TvP	CV%	*ω*
K_a_ (1/h)	0.21	7.52	0.015
V (ml/kg)	447.58	41.95	0.474
K_m_ (ng/ml)	237.60	23.85	0.041
V_max_ (ng/h/kg)	1,252,830	14.20	—
Res. Error	1.04	6.81	—

CV%, coefficient of variation; Res. Error, residual error.

The mean V of the population was estimated to be 447.58 ml/kg, suggesting that the distribution of HY-021068 mainly in the body fluid of beagle dogs. The V_max_ and K_m_ were estimated to be 1,252,830 ng/h/kg and 237.60 ng/ml, which indicated high dose group was more easily to be affected by nonlinear elimination.

### Pharmacodynamics

Both 2.5 and 5 mg/kg oral administration of HY-021068 significantly inhibited the aggregation of platelet. The PAR was about 55% at baseline (pre-dose), which reduced to 31.4 and 26.4% after oral administration of 2.5 and 5 mg/kg HY-021068.2.5 mg/kg HY-021068 can achieve similar minimum PAR with aspirin (HY-021068 vs aspirin: 31.4 ± 7.1% vs. 33.6 ± 10.3%) (Figure 3B). Model fitting and observed PAR after single oral 2.5 and 5 mg/kg HY-021068 administration were displayed in Figure 3C and parameter estimates were listed in Table 4. The inter-individual variability (*ω*) with high shrinkage value (>0.4) was excluded from the PD model to avoid over-parameterization.

**TABLE 4:** Population PD results of oral administration of HY-021068 to beagle dogs.

Parameter	TvP	CV%	*ω*
β	0.475	3.11	—
K_syn_ (ng/h)	88.501	2.54	—
K_deg_ (1/h)	0.515	28.36	0.466
K (ml/ng/h)	0.002	24.96	0.775
MultStdev	0.122	7.27	—
Res. Error	0.003	4.49	—

CV%, coefficient of variation; Res. Error, residual error.

### Model Evaluation and Validation

**FIGURE 4:**
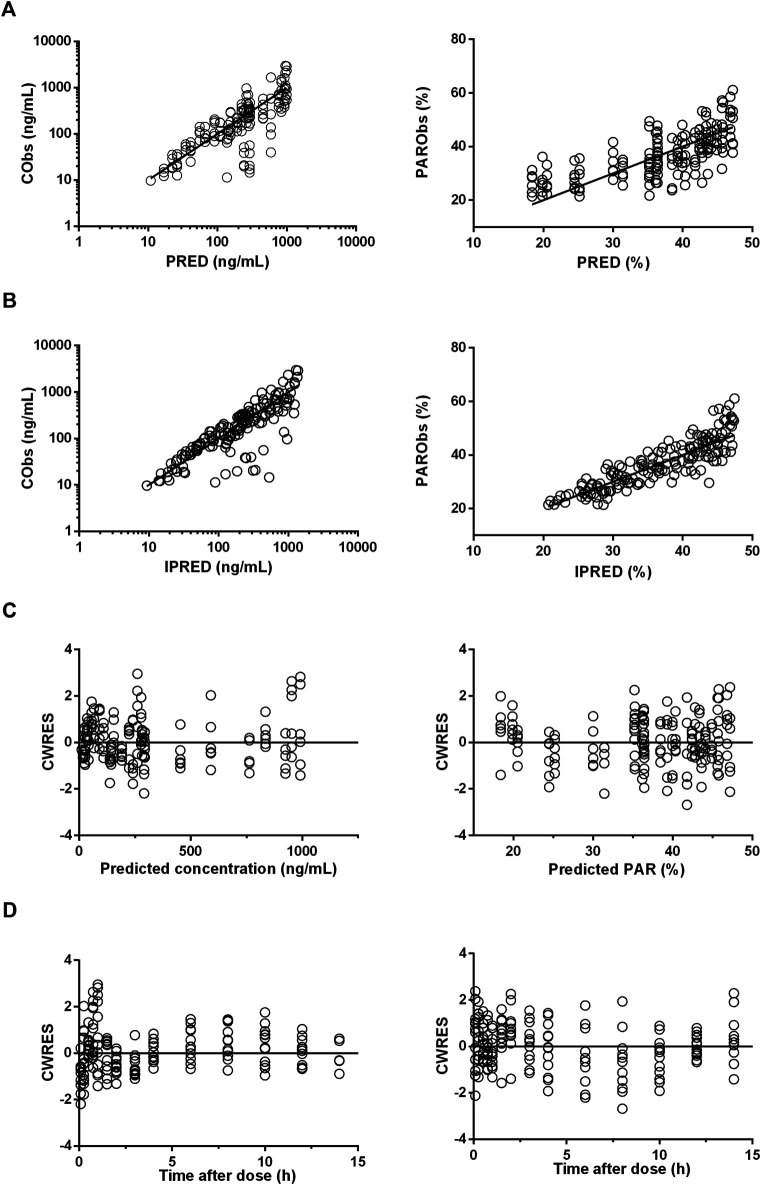
Diagnostic plots for pharmacokinetic (PK) (left panels) and pharmacodynamic (PD) (right panels) profile estimations. **(A)**, The observed values (Obs) vs. population predicted values (PRED). **(B)**, The observed values vs. individual predicted values (IPRED). **(C)**, CWRES vs. population predictions. **(D)**, CWRES vs. time after dose.

**FIGURE 5:**
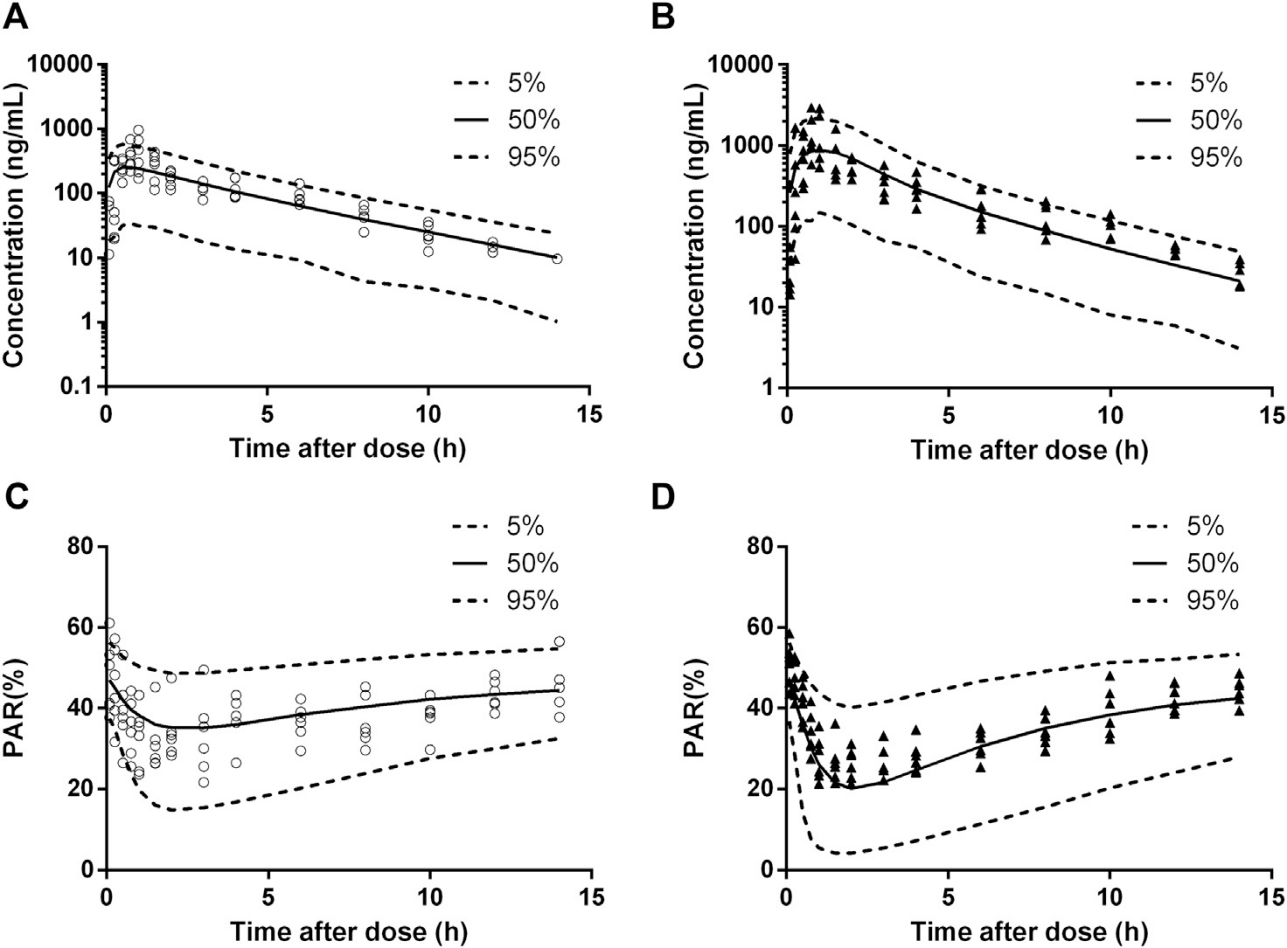
Visual prediction check for pharmacokinetic (PK) and pharmacodynamic (PD) estimation after a single oral dose of 2.5 mg/kg (cicles, n = 6) and the first administration of multiple doses of 5 mg/kg (triangles, n = 6). **(A),** 2.5 mg/kg dosing for PK. **(B),** 5 mg/kg dosing for PK. **(C),** 2.5 mg/kg dosing for PD. **(D),** 5 mg/kg dosing for PD. Solid and dashed lines depict median and 90% CI of model predictions.

**FIGURE 6:**
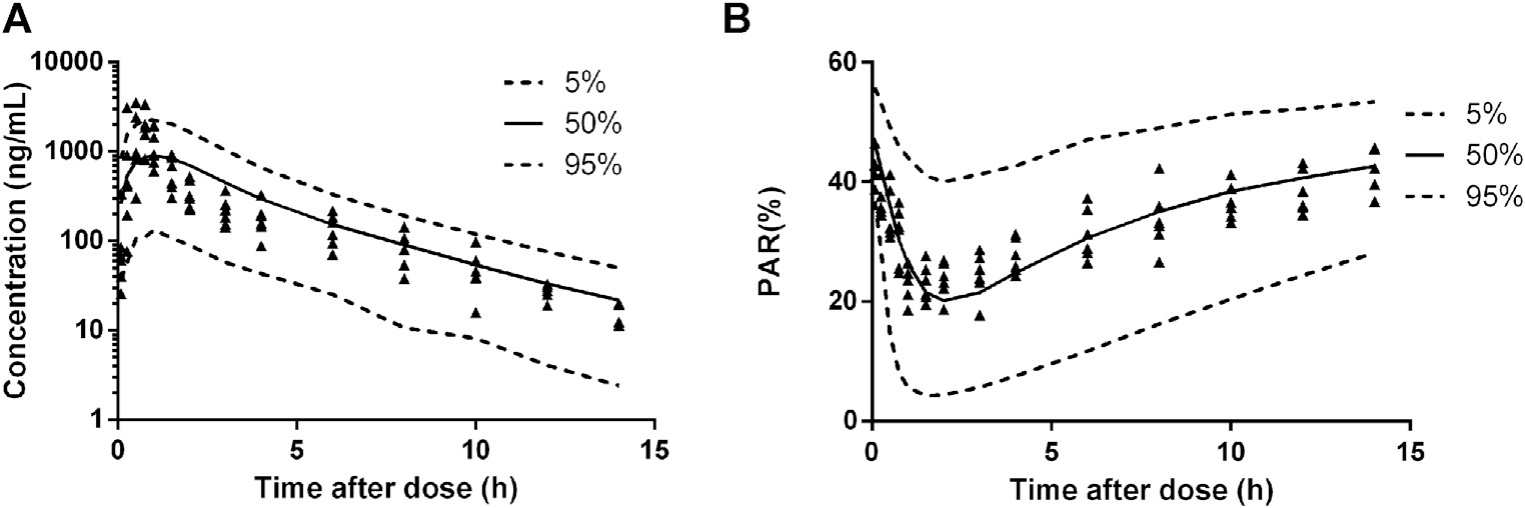
Predicted plasma HY-021068 concentration-time profiles **(A)** and PAR **(B)** in beagle dogs on the 7th day following oral 5 mg/kg administration for consecutive 7 days (n = 6). Solid and dashed lines depict median and 90% CI of model predictions.

The diagnostic plots of the PK-PD model were shown in Figure 4. The population-predicted and individual predicted concentration and PAR vs observed values indicated no obvious bias. The scatter plot of CWRES vs predicted values or time after dose (TAD) for PK and PD data showed most conditional weighted residual lay within the range of −2 to 2. The bias for a few concentrations and PAR may due to the variability of C_max_ and determination of efficacy index. Visual prediction check for 2.5 and 5 mg/kg showed almost all observed concentrations and PAR fell into the 5th and 95th percentiles of the simulated data (Figure 5). An internal-external validation model was applied (Steyerberg and Harrell, 2015). Simulated results showed that the predicted concentrations and PAR matched well with the observed values on the 7th day after 5 mg/kg dosing for consecutive 7 days (Figure 6). No cumulative effect was observed. All of these indicated our model is reasonable and powerful.

## Discussion

In this study, a semi-mechanistic PK/PD model based on the irreversible inhibitory of TXS was successfully constructed to model the time course of plasma concentrations of HY-021068 and the PAR.

Many models aimed to depict the time course of PAR have been established before (Awa et al., 2012; Yun et al., 2014). However, this is the first PK-PD model in which irreversible combination of drugs and TXS is considered. By comparing the objective function value under different PK models, the PK of HY-021068 was depicted best using a one-compartment model with first-order absorption and saturated elimination. The population typical values and relative standard errors of K_a_, V, K_m_ and V_max_ for HY-021068 were 0.211 1/h (7.52%), 447.579 ml/kg (41.95%), 237.603 ng/ml (23.85%), 1,252,830 ng/h/kg (14.2%), respectively. This showed the main inter-individual variability of PK data may be due to the differences in apparent volume of distribution among beagle dogs.

Many reports have also demonstrated that platelet activity can be reduced by inhibiting the formation of TXA_2_ (Banerjee et al., 2014; Fang et al., 2014). AA was widely used as platelet agonists (Pyo et al., 2007; Andrioli et al., 2015; Olivier et al., 2016) in the platelet aggregation test. HY-021068 targeted the TXS on the AA pathway and inhibited the platelet aggregation by reducing the transformation of AA to TXA_2_ (Yang et al., 2015). No active metabolites of HY-021068 were found in previous studies, so anti-platelet activity was only influenced by the parent drug. The indirect effect model and irreversible model were tried to fit the relationship between plasma concentrations and PAR. Although both models showed similar goodness-of-fit during the model fitting process, the irreversible model showed better predictive ability when extrapolated to the multiple dose administration. This also hinted that the underlying binding pattern of HY-021068 and TXS was probably irreversible, which still remains unclear. The population typical values and relative standard errors of *β*, K_syn_, K_deg_ and K were 0.475 (3.11%), 88.501 ng/h (2.54%), 0.515 1/h (28.36%) and 0.002 ml/ng/h (24.96%), respectively. The inter-individual variability of PD data may be linked to the differences in the degradation rate of TXS as well as the affinity between HY-021068 and TXS among beagle dogs.

A limitation of the present study was that the synthesis and degradation rate of TXS in beagle dogs have not been reported before, so we obtained the parameters according to the estimate of the model. If the related physiological parameters are reported in the future, the K_syn_ and K_deg_ can be fixed before the run of the PD model. It may be more related to the physical conditions with a minor modification of the present model. Additionally, the structure of the model may be further utilized in clinical experiments in the future.

In conclusion, this study established a mechanism-based PK/PD model for characterizing the pharmacokinetics of HY-021068 and its effect in beagle dogs. The integrated PK/PD model enabled the quantitative description of the relationship between the HY-021068 plasma concentration and its inhibitory effect on platelet aggregation based on the drug pharmacological mechanisms. The final model can be subsequently used to predict the concentration and corresponding effect under different dosages. It provides reference to other drugs that share a similar mechanism with HY-021068. It also offers a fundamental PK/PD model that could be used in humans.

## Data Availability Statement

The raw data supporting the conclusions of this article will be made available by the authors, without undue reservation.

## Ethics Statement

The animal study was reviewed and approved by Animal Ethics Committee of China Pharmaceutical University.

## Author Contributions

PL, JH, JZ, GY, LL participated in research design. DG, PHL, ZC, PL conducted experiments. PL, JH, DG performed data analysis. PL and LL wrote the manuscript.

## Funding

This work was supported by the National Natural Science Foundation of China (No. 81673505 and 81872930) and the “Double First-Class” University project (No. CPU2018GY22).

## Conflict of Interest

The authors declare that the research was conducted in the absence of any commercial or financial relationships that could be construed as a potential conflict of interest.
